# Supplementary figures and images for: Polymorphisms in the hypoxia inducible factor binding site of the macrophage migration inhibitory factor gene promoter in schizophrenia
Source: PLoS One. 2022 Mar 24;17(3):e0265738. doi: 10.1371/journal.pone.0265738 (PMC8946738; doi:10.1371/journal.pone.0265738)

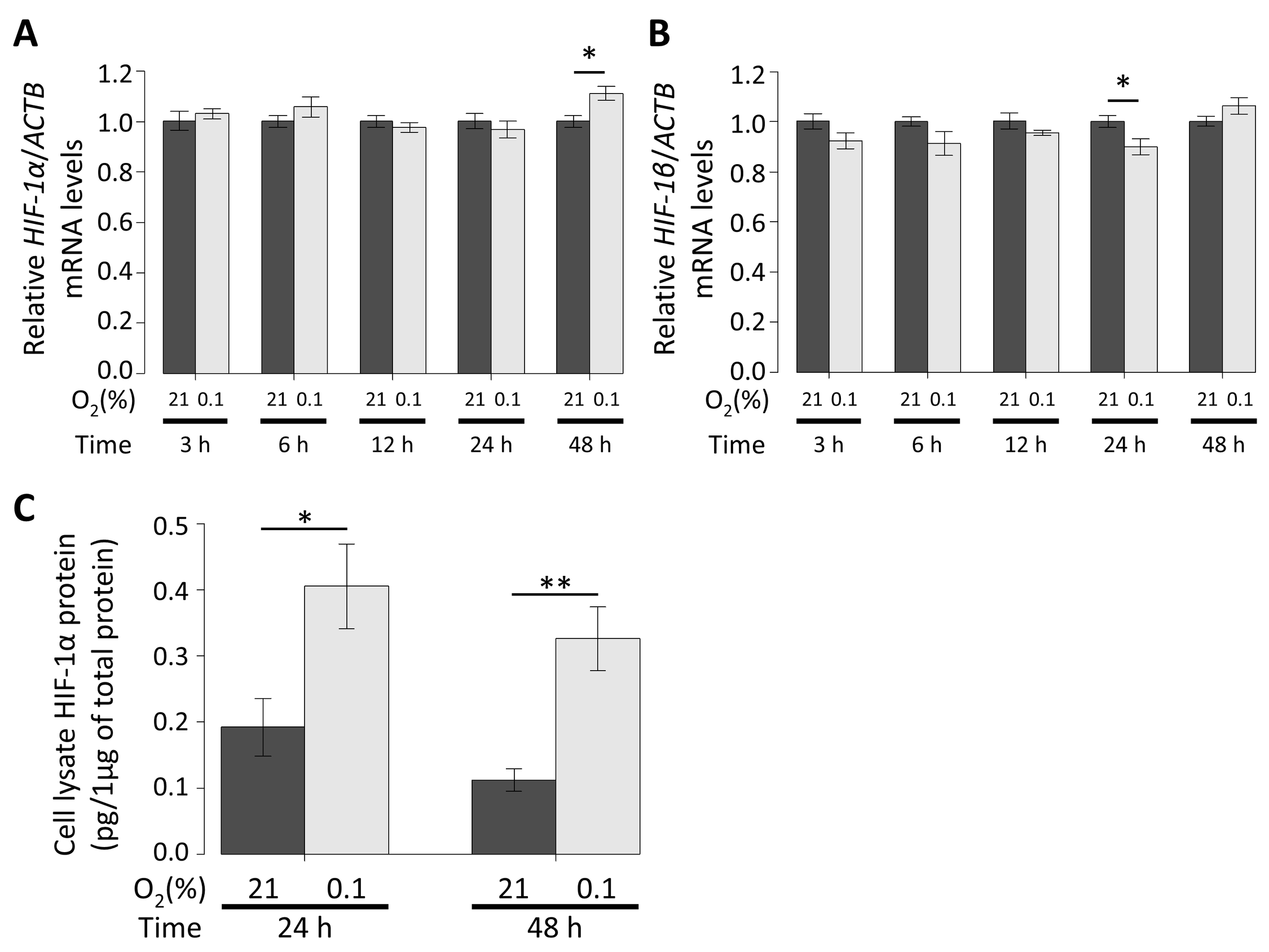

Supplement: S1 Fig — A. The time-dependent effects of hypoxia on HIF-1α mRNA expression in primary cultured astrocytes (PCAs). PCAs were incubated for 3, 6, 12, 24, or 48 h in 21.0% or 0.1% O2. HIF-1α mRNA expression was analyzed by qRT-PCR. The values are shown as the ratio of HIF-1α mRNA to beta-actin (ACTB) mRNA (Student’s t-test; n = 6). B. The time-dependent effects of hypoxia on HIF-1β mRNA expression in PCAs. PCAs were incubated for 3, 6, 12, 24, or 48 h in 21.0% or 0.1% O2. HIF-1β mRNA expression was analyzed by qRT-PCR. The values are shown as the ratio of HIF-1β mRNA to ACTB mRNA (Student’s t-test; n = 6). C. The effects of hypoxia on HIF-1α protein level in cell lysate of PCAs. PCAs were incubated for 24 or 48 h in 21.0% or 0.1% O2. The HIF-1α protein level per 1 μg of total protein was analyzed using a HIF-1α enzyme-linked immunosorbent assay (ELISA) (Student’s t-test; n = 6). The data are expressed as the mean ± SEM. *p < 0.05, **p < 0.01, or ***p < 0.001. (TIF) [file pone.0265738.s001.tif]

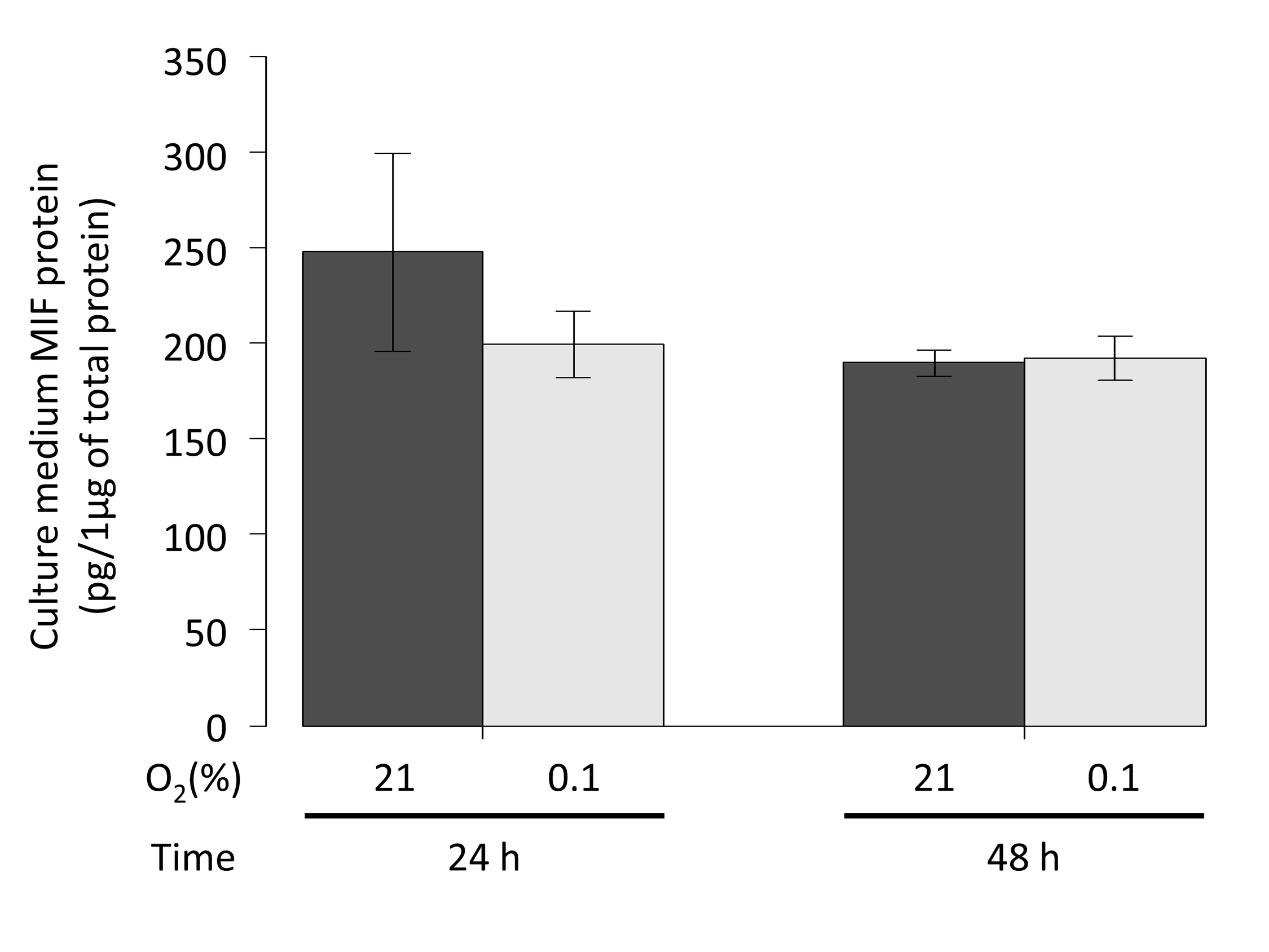

Supplement: S2 Fig — PCAs were incubated for 24 or 48 h in 21.0% or 0.1% O2. The MIF protein level per 1 μg of total protein was analyzed using a MIF enzyme-linked immunosorbent assay (ELISA). The data are expressed as the mean ± SEM. *p < 0.05, **p < 0.01, or ***p < 0.001 (Student’s t-test; n = 6). (TIF) [file pone.0265738.s002.tif]
