# Supplementary material for: Polymorphisms in the hypoxia inducible factor binding site of the macrophage migration inhibitory factor gene promoter in schizophrenia
Source: PLoS One. 2022 Mar 24;17(3):e0265738. doi: 10.1371/journal.pone.0265738 (PMC8946738; doi:10.1371/journal.pone.0265738)
Supplement: S1 Table — (DOCX) [file pone.0265738.s003.docx]

**S1 Table. Demographic and clinical characteristics of participants for the macrophage migration inhibitory factor gene polymorphism association study.**

|  | First set |  |  |  | Second set |  |  |
| --- | --- | --- | --- | --- | --- | --- | --- |
|  | Control | Schizophrenia | *P*-value |  | Control | Schizophrenia | *P*-value |
| Number | 836 | 915 |  |  | 671 | 843 |  |
| Sex (male/female) | 398/438 | 493/422 | 0.00975^a^ |  | 341/330 | 453/390 | 0.259^a^ |
| Age (years; mean ± SD) | 52.5 ± 18.7 | 54.2 ± 15.1 | 0.0398^b^ |  | 38.3 ± 10.8 | 40.8 ± 13.8 | 0.000112^b^ |
| Age of onset (years; mean ± SD) | - | 26.1 ± 8.9 |  |  | - | 23.0 ± 7.4 |  |
| Duration of illness (years; mean ± SD) | - | 27.6 ± 14.6 |  |  | - | 17.5 ± 12.3 |  |

Abbreviation: SD, standard deviation.

^a^ *P*-value was calculated with the χ^2^ test between the schizophrenia and control groups.

^b^ *P*-value was calculated with Student’s t-test between the schizophrenia and control groups.
