# Supplementary material for: Polymorphisms in the hypoxia inducible factor binding site of the macrophage migration inhibitory factor gene promoter in schizophrenia
Source: PLoS One. 2022 Mar 24;17(3):e0265738. doi: 10.1371/journal.pone.0265738 (PMC8946738; doi:10.1371/journal.pone.0265738)
Supplement: S2 Table — (DOCX) [file pone.0265738.s004.docx]

**S2 Table. Primers used in the experiments with primary cultured astrocytes from mouse.**

| Designation | Sequence |
| --- | --- |
| qRT-PCR mouse ACTB Fwd | ACTGCTCTGGCTCCTAGCAC |
| qRT-PCR mouse ACTB Rev | CCACCGATCCACACAGAGTA |
| qRT-PCR mouse HIF-1α Fwd | AACAGAATGGAACGGAGCAA |
| qRT-PCR mouse HIF-1α Rev | TTCACAATCGTAACTGGTCAGC |
| qRT-PCR mouse HIF-1β Fwd | TGCCTCATCTGGTACTGCTG |
| qRT-PCR mouse HIF-1β Rev | TGTCCTGTGGTCTGTCCAGT |
| qRT-PCR mouse MIF Fwd | CCCAGAACCGCAACTACAG |
| qRT-PCR mouse MIF Rev | GCAGCGTTCATGTCGTAATAGT |
| ChIP mouse MIF Fwd | ATGTAATACTTCCTACAGCACCAGAAG |
| ChIP mouse MIF Rev | CTACGTGACCCAGCTCAGTACC |
| Infusion mouse MIF Fwd | ACTGGCCGGTACCTGTATACGGTTAATCTGTAGCAT |
| Infusion mouse MIF Rev | CCGGATTGCCAAGCTGGTGGCGGAGAGACTGCG |
| snpHRE mouse MIF Fwd | AACGTAGCTCAGGTCCCTGGCTTGGGTCA |
| snpHRE mouse MIF Rev | ACCCAGCTCAGTACCGCCCCAA |
| mutHRE mouse MIF Fwd | CTAGCAGCTCAGGTCCCTGGCTTGGGTCA |
| mutHRE mouse MIF Rev | ACCCAGCTCAGTACCGCCCCAA |
| delHRE mouse MIF Fwd | AGCTCAGGTCCCTGGCTTGGGTCA |
| delHRE mouse MIF Rev | ACCCAGCTCAGTACCGCCCCAA |

Abbreviation: ACTB, beta-actin; ChIP, chromatin immunoprecipitation; Fwd, forward; HIF, hypoxia inducible factor; HRE, hypoxia response element; MIF, macrophage migration inhibitory factor; qRT-PCR, quantitative reverse transcription polymerase chain reaction; Rev, reverse.

The primers harboring the desired mutations, such as SNP, mutation, and deletion versions of the HRE, are shown as snpHRE, mutHRE, and delHRE, respectively.
