# Supplementary material for: Polymorphisms in the hypoxia inducible factor binding site of the macrophage migration inhibitory factor gene promoter in schizophrenia
Source: PLoS One. 2022 Mar 24;17(3):e0265738. doi: 10.1371/journal.pone.0265738 (PMC8946738; doi:10.1371/journal.pone.0265738)
Supplement: S3 Table — (DOCX) [file pone.0265738.s005.docx]

**S3 Table. Distribution of rs17004038 in patients with schizophrenia and controls in the first set of subjects.**

|  |  |  | Genotype | |  |  |  | Allele |  |  |  |  |  |
| --- | --- | --- | --- | --- | --- | --- | --- | --- | --- | --- | --- | --- | --- |
|  | n | HWE | C/C | C/A | A/A | *P*-value^a^ |  | C | A | MAF | *P*-value^b^ | Odds ratio (95% CI) | Power |
| Overall |  |  |  |  |  |  |  |  |  |  |  |  |  |
| SCZ | 915 | 1.00 | 865 | 49 | 1 | 0.0839 |  | 1779 | 51 | 0.0279 | 0.0509 | 1.569 (0.995-2.475) | 0.503 |
| CTL | 836 | 1.00 | 806 | 30 | 0 |  |  | 1642 | 30 | 0.0179 |  |  |  |
| Male |  |  |  |  |  |  |  |  |  |  |  |  |  |
| SCZ | 493 | 0.61 | 467 | 25 | 1 | 0.462 |  | 959 | 27 | 0.0274 | 0.238 | 1.466 (0.774-2.775) | 0.217 |
| CTL | 398 | 1.00 | 383 | 15 | 0 |  |  | 781 | 15 | 0.0188 |  |  |  |
| Female |  |  |  |  |  |  |  |  |  |  |  |  |  |
| SCZ | 422 | 1.00 | 398 | 24 | 0 | 0.140 |  | 820 | 24 | 0.0284 | 0.115 | 1.680 (0.875-3.225) | 0.351 |
| CTL | 438 | 1.00 | 423 | 15 | 0 |  |  | 861 | 15 | 0.0171 |  |  |  |

Abbreviations: CI, Confidence interval; CTL, control; HWE, Hardy-Weinberg equilibrium; MAF, minor allele frequency; SCZ, schizophrenia.

^a^ Genotypic p-values were calculated with Cochran-Armitage trend test.

^b^ Allelic p-values were calculated with the χ^2^ test.
