# Supplementary material for: Polymorphisms in the hypoxia inducible factor binding site of the macrophage migration inhibitory factor gene promoter in schizophrenia
Source: PLoS One. 2022 Mar 24;17(3):e0265738. doi: 10.1371/journal.pone.0265738 (PMC8946738; doi:10.1371/journal.pone.0265738)
Supplement: S4 Table — (DOCX) [file pone.0265738.s006.docx]

**S4 Table. Distribution of rs17004038 in patients with schizophrenia and controls in the second set of subjects.**

|  |  |  | Genotype | |  |  |  | Allele |  |  |  |  |  |
| --- | --- | --- | --- | --- | --- | --- | --- | --- | --- | --- | --- | --- | --- |
|  | n | HWE | C/C | C/A | A/A | *P*-value^a^ |  | C | A | MAF | *P*-value^b^ | Odds ratio (95% CI) | Power |
| Overall |  |  |  |  |  |  |  |  |  |  |  |  |  |
| SCZ | 843 | 1.00 | 812 | 31 | 0 | 0.388 |  | 1655 | 31 | 0.0184 | 0.364 | 1.304 (0.733-2.319) | 0.143 |
| CTL | 671 | 3.0×10^−4^ | 655 | 13 | 3 |  |  | 1323 | 19 | 0.0142 |  |  |  |
| Male |  |  |  |  |  |  |  |  |  |  |  |  |  |
| SCZ | 453 | 1.00 | 437 | 16 | 0 | 0.993 |  | 890 | 16 | 0.0177 | 0.992 | 1.004 (0.472-2.136) | 0.050 |
| CTL | 341 | 8.7×10^−5^ | 332 | 6 | 3 |  |  | 670 | 12 | 0.0176 |  |  |  |
| Female |  |  |  |  |  |  |  |  |  |  |  |  |  |
| SCZ | 390 | 1.00 | 375 | 15 | 0 | 0.180 |  | 765 | 15 | 0.0192 | 0.184 | 1.829 (0.741-4.513) | 0.258 |
| CTL | 330 | 1.00 | 323 | 7 | 0 |  |  | 653 | 7 | 0.0106 |  |  |  |

Abbreviations: CI, Confidence interval; CTL, control; HWE, Hardy-Weinberg equilibrium; MAF, minor allele frequency; SCZ, schizophrenia.

^a^ Genotypic p-values were calculated with Cochran-Armitage trend test.

^b^ Allelic p-values were calculated with the χ^2^ test.
