# Supplementary material for: Polymorphisms in the hypoxia inducible factor binding site of the macrophage migration inhibitory factor gene promoter in schizophrenia
Source: PLoS One. 2022 Mar 24;17(3):e0265738. doi: 10.1371/journal.pone.0265738 (PMC8946738; doi:10.1371/journal.pone.0265738)
Supplement: S5 Table — (DOCX) [file pone.0265738.s007.docx]

**S5 Table. Allele frequency of SNP rs17004038 in different ethnic populations.**

| Populations |  | C/A counts | A allele frequency |
| --- | --- | --- | --- |
| Global |  | 4986/22 | 0.0044 |
| ACB | African Carribbeans in Barbados | 192/0 | 0.0000 |
| ASW | Americans of African ancestry in SW, USA | 122/0 | 0.0000 |
| BEB | Bengali from Bangladesh | 172/0 | 0.0000 |
| CDX | Chinese Dai in Xishuangbanna, China | 181/5 | 0.0269 |
| CEU | Utah residents with North and Western European ancestry | 198/0 | 0.0000 |
| CHB | Han Chinese in Beijing, China | 203/3 | 0.0146 |
| CHS | Han Chinese South, China | 203/7 | 0.0333 |
| CLM | Colombians from Medellin, Colombia | 188/0 | 0.0000 |
| ESN | Esan in Nigeria | 198/0 | 0.0000 |
| FIN | Finnish in Finland | 198/0 | 0.0000 |
| GBR | British in England and Scotland, UK | 182/0 | 0.0000 |
| GIH | Gujarati Indian from Houston, Texas | 206/0 | 0.0000 |
| GWD | Gambian in Western Divisions in Gambia | 226/0 | 0.0000 |
| IBS | Iberian populations in Spain | 214/0 | 0.0000 |
| ITU | Indian Telugu from the UK | 204/0 | 0.0000 |
| JPT | Japanese in Tokyo, Japan | 203/5 | 0.0240 |
| KHV | Kinh in ho Chi Minh City, Vietnam | 196/2 | 0.0101 |
| LWK | Luhya in Webuye, Kenya | 198/0 | 0.0000 |
| MSL | Mende in Sierra Leone | 170/0 | 0.0000 |
| MXL | Mexican ancestry from Los Angeles, USA | 128/0 | 0.0000 |
| PEL | Peruvians from Lima, Peru | 170/0 | 0.0000 |
| PJL | Punjabi from Lahore, Pakistan | 192/0 | 0.0000 |
| PUR | Puerto Ricans in Puerto Rico | 208/0 | 0.0000 |
| STU | Sri Lankan Tamil from the UK | 204/0 | 0.0000 |
| TSI | Toscani in Italia | 214/0 | 0.0000 |
| YRI | Yoruba in Ibadan, Nigeria | 216/0 | 0.0000 |

The data for allele frequency were derived from 1000 Genomes Project Data Phase 3 (https://www.ncbi.nlm.nih.gov/variation/tools/1000genomes/).
